# Brazilian Registry of Cardiovascular Surgery in Adults Fully Operational

**DOI:** 10.5935/1678-9741.20160040

**Published:** 2016

**Authors:** Walter J. Gomes, Renato A. K. Kalil, Fabio B. Jatene

**Affiliations:** 1 Escola Paulista de Medicina, Universidade Federal de São Paulo (UNIFESP), São Paulo, SP, Brazil.; 2 Instituto de Cardiologia do Rio Grande do Sul, Fundação Universitária de Cardiologia, Porto Alegre, RS, Brazil.; 3 Instituto do Coração do Hospital das Clínicas da Faculdade de Medicina da Universidade de São Paulo (InCor HC-FMUSP), São Paulo, SP, Brazil.

The Sociedade Brasileira de Cirurgia Cardiovascular (SBCCV) has taken great pleasure and pride in announcing that the Brazilian Registry of Cardiovascular Surgery in Adults (The Bypass Registry) is currently fully operational and the inclusion has exceeded 1500 patients in the first nine months of operation. This success is the aftermath of the serious, diligent and willful work of the Brazilian cardiovascular surgeons involved in the project.

The establishment of the Brazilian Registry of Cardiovascular Surgery in Adults sets a long-standing need for fundamental understanding of the real figures for the country pertaining to the cardiovascular surgery practice and as a result to develop strategies for improvements in quality and excellence.

The Project is an enterprise of the SBCCV along with the HCor Research Institute and aims to document the practice of cardiovascular surgeries throughout the national territory, in centers of all Brazilian states, including public (university and nonuniversity) and private hospitals. Surgical procedures included in the registry encompass coronary artery bypass surgery, valve surgery, aortic surgery, atrial fibrillation procedures, heart transplantation, mechanical circulatory support, and congenital heart defects in adults.

With the SBCCV increasingly being introduced to the international community of cardiovascular surgery and being recognized, steps still need to be traversed in order to reach the quality standards of first world countries.

The Registry is a database designed to collect a multiplicity of clinical parameters of patients undergoing cardiovascular surgery in Brazil, and uniquely this study will followed up late the patients, from discharge extending for assessment in 30 days, 6 months and 12 months to evaluate cardiovascular events in the medium- and long-term.

The project also will require effort, goodwill and willingness of the participating centers and those in charge of data collection, since, currently, the project is funded solely by SBCCV and consequently budget constraints apply.

The stored data is a classified information (only the institution itself is to have access to their own data) and analyzed together to develop best practices in cardiovascular surgery in Brazilian hospitals. It will be examined to plan future improvement of clinical practice programs, improve quality, optimize outcomes and serve as a base for clinical studies, to determine resource utilization, and others. Such information is not available today in a database that includes all kind of patients, since the only available database is that of the public health system. We hope that this could provide basis for health policy planning at local, regional and national levels.

Data from new centers are to be added, as the inclusion process is open and active. Our initial fourteen centers are expanding and we reiterate the invitation to centers from all over the country to join and participate in this endeavor. Come be part of the BYPASS PROJECT.

Thank you all.

